# The phospholipase D inhibitor FIPI potently blocks EGF-induced calcium signaling in human breast cancer cells

**DOI:** 10.1186/s12964-021-00724-z

**Published:** 2021-04-08

**Authors:** Helena M. Stricker, Nadine Rommerswinkel, Silvia Keil, Sandina A. Gnoth, Bernd Niggemann, Thomas Dittmar

**Affiliations:** 1 Institute of Immunology, Center of Biomedical Education and Research (ZBAF), Witten/Herdecke University, Witten, Germany; 2 Community Hospital Herdecke, Herdecke, Germany; 3 Ruhr-University Bochum, Bochum, Germany

**Keywords:** Breast cancer, PLD, PLC-γ1, Cell migration

## Abstract

**Background:**

Phosphotyrosine kinase (PTK)-mediated phospholipase C-γ1 (PLC-γ1) signaling plays a crucial role in the release of the universal second messenger calcium from intracellular stores, which is mandatory for several cellular processes, including cell migration. However, PLC-γ1 could also be activated in a PTK-independent manner by phospholipase D (PLD)-derived phosphatidic acid (PA). Because both higher PLD expression levels and PLD activity have also been associated with breast cancer cell invasion and migration, we wondered whether there might be a link between PLD and PLC-γ1, which was investigated in this study.

**Materials:**

MDA-MB-468-NEO (EGFR positive) and MDA-MB-468-HER2 (EGFR and HER2 positive) human breast cancer cells were used in this study. The migratory behavior of the cells in the presence of epidermal growth factor (EGF) and the PLD inhibitor 5-fluoro-2-indolyl-des-chlorohalopemide (FIPI) was analyzed using the 3D collagen matrix migration assay. Changes in cytosolic calcium levels in the presence of EGF, FIPI and Sig-1R agonists and antagonists as well as in PLD1 siRNA knockdown cells were determined by flow cytometry. Western blot analyses were performed to determine the basal expression levels and phosphorylation patterns of EGFR, HER2, AKT, MAPK^p42/44^, PLC-γ1 and Sig-1R.

**Results:**

The EGF-induced migration of MDA-MB-468-NEO and MDA-MB-468-HER2 cells was significantly impaired by FIPI. Likewise, FIPI also significantly abolished EGF-induced calcium release in both cell lines. However, neither the expression levels nor the phosphorylation patterns of EGFR, HER2, AKT, MAPK^p42/44^ and PLC-γ1 were markedly changed by FIPI. Knockdown of PLD1 expression by siRNA also significantly impaired EGF-induced calcium release in both cell lines. Targeting Sig-1R, which interacts with IP3R, with the antagonist BD1047 also abrogated EGF-induced calcium release. However, EGF-induced calcium release was also impaired if cells were treated with the Sig-1R agonists PRE084 and PPBP maleate.

**Conclusion:**

In summary, blocking PLD activity with the specific inhibitor FIPI or knocking down PDL1 expression by siRNA significantly impaired EGF-induced calcium release in MDA-MB-468-NEO and MDA-MB-468-HER2 cells, likely indicating a connection between PLD activity and PLC-γ1-mediated calcium signaling. However, how PLD activity interferes with the release of calcium from intracellular stores remains unclear.

**Video Abstract**

**Supplementary Information:**

The online version contains supplementary material available at 10.1186/s12964-021-00724-z.

## Background

It is well recognized that receptor tyrosine kinase (RTK)-mediated activation of phospholipase C-γ1 (PLC-γ1) plays a crucial role in the release of universal second messengers from intracellular stores, such as the endoplasmic reticulum and sarcoplasmic reticulum [1, 28, 51]. PLC-γ1 belongs to the large family of phosphoinositide-specific PLCs, which can be subdivided into six subfamilies (β, γ, δ, ε, η, and ζ) [1, 28]. All isoforms have in common that they catalyze the hydrolysis of phosphatidylinositol-4,5-bisphosphate (PIP2) to inositol-1,4–5-triphosphate (IP3) and diacylglycerol (DAG) in response to the activation of more than 100 different cell surface receptors, including RTKs and G-protein coupled 7-transmembrane receptors (GPCRs) [51]. The binding of IP3 to inositol-1,4,5-trisphosphate receptor (IP3R), which is localized in the membranes of the endoplasmic and sarcoplasmic reticulum, results in increased cytosolic calcium levels [28, 51]. In contrast, DAG remains in the plasma membrane of the cells and is mandatory for subsequent PKC activation [28, 51].

To date, six phospholipase D (PLD) isoforms (PLD1 to PLD6) have been identified in humans, whereby PLD1 and PLD2 are still the best-characterized members of the PLD family [17, 45, 47]. PLD1 and PLD2 are so-called ‘classical’ PLDs since they convert phosphatidylcholine to choline and phosphatidic acid (PA), whereas PLD3-6 lack this PLD activity and hence have been named nonclassical PLDs [17, 45, 47]. The isoforms PLD3 and PLD4 are single-stranded acid exonucleases anchored in the membrane of the endoplasmic reticulum via a transmembrane domain and regulate endosomal nucleic acid sensing [15]. Dysfunction/mutation of PLD3 and PLD4, respectively, have been associated with Alzheimer’s disease (PLD3) and rheumatoid arthritis and systemic sclerosis (PLD4) [15, 47]. The function of PLD5 remains unknown [45, 47], and PLD6, which is bound to the outer mitochondrial membrane via a transmembrane tail, is rather an RNase and involved in the generation of piwi-interacting RNAs [1, 45, 47].

PLD1/2 can be activated via different G-protein coupled receptor (GPCR)-[8, 37] and receptor tyrosine kinase (RTK)-induced signal transduction cascades [24, 25, 38, 46, 57], including Ras-RalA signaling [42, 63] and PKC signaling [14, 25, 35, 60]. Additionally, PLD1 could also be directly activated via the EGFR adaptor protein FAM83B [10]. It is well known that PLD1/2 activity and its product PA are involved in various cellular processes, including proliferation, survival, membrane trafficking, autophagy, and cell migration [1, 18, 22, 26, 31, 36, 45, 47]. For instance, Kantonen and colleagues demonstrated that leukocyte phagocytosis is regulated by the interplay of PLD2, WASP, and Grb2 [31], which might also play a role in EGF- and colony stimulating factor-1 (CSF-1)-induced chemotaxis of fibroblasts and macrophages [36]. Koch et al. concluded from their data that PLD2 plays an essential role in the regulation of agonist-dependent and agonist-independent GPCR endocytosis [37]. Furthermore, it was shown that PLD-derived PA could promote cell migration through Fer-induced actin polymerization [26]. A similar mechanism has been proposed for macrophages and fibroblasts, whereby PLD-derived PA interacts with the mammalian target of rapamycin (mTOR) and S6-kinase (S6K), thereby promoting reorganization of the actin cytoskeleton and cell migration [36].

In addition to physiological processes, aberrant PLD expression levels and increased PLD activity have also been associated with cancer development and progression [10, 11, 22, 23, 29, 30, 41, 43, 66]. Kang and colleagues concluded from their findings that the invasion of breast cancer cells was related to both higher PLD1 expression levels and increased PLD1 activity [29, 30]. In contrast, Henkels et al. showed that invasion, growth, and metastatic spreading of breast cancer cells in a xenograph model were rather PLD2 dependent [22, 23]. Overall survival analyses suggested that high expression levels of stearoyl-CoA desaturase-1 (SCD1) and PLD2 in breast cancer patients were both associated with metastasis-related morbid outcomes [41]. Therefore, in vitro studies revealed that cell migration properties were regulated by SCD1 activity through a PLD-mTOR/S6K signaling pathway [41]. However, Diaz-Aragon and colleagues concluded from their data that both PLD1 and PLD2 were involved in the linoleic acid-induced migration and invasion as well as sphere formation of MDA-MB-231 human breast cancer cells [11].

Due to the involvement of PLD in breast cancer migration and invasion [11, 22, 30, 41], we wondered whether PLD activity might also play a role in the migration of MDA-MB-468-NEO (MDA-NEO) and MDA-MB-468-HER2 (MDA-HER2) human breast cancer cells [3, 9, 12, 52, 53]. Previous studies revealed that both breast cancer cell lines differed not only in their mode of calcium oscillations but also in their susceptibility to the PLC-γ1 inhibitor U73122 [3, 12]. A concentration of 2 µM U73122 was sufficient to completely inhibit EGF-induced calcium signaling in MDA-HER2 cells. In contrast, up to 10 µM U73122 was necessary to block EGF-induced calcium signaling in MDA-NEO cells [12], suggesting that more active PLC-γ1 molecules were present in the MDA-NEO cell line than in MDA-HER2 cells. Given that PLC-γ1 could also be activated by PLD-derived PA [51], we wondered whether the inhibition of PLD activity might also have an impact on PLC-γ1 activity and PLC-γ1-related calcium signaling.

## Methods

### Cell culture

MDA-NEO and MDA-HER2 breast cancer cells were derived from MDA-MB-468 breast cancer cells (HTB-132; LGC Standards GmbH, Wesel, Germany) by stable transduction with a G418 resistant vector (MDA-NEO) or a HER2 (c-erbB-2) expression vector (MDA-HER2) [3, 12, 52, 53]. Cells were cultured in DMEM (Sigma-Aldrich, Taufkirchen, Germany) supplemented with 10% FCS (Biochrom GmbH, Berlin, Germany), 10 U/ml penicillin, 10 mg/ml streptomycin (Sigma-Aldrich, Taufkirchen, Germany), and 400 µg/ml G418 (Biochrom GmbH, Berlin, Germany) in a humidified atmosphere at 37 °C and 5% CO_2_.

### Cell migration studies

The migration of MDA-NEO and MDA-HER2 breast cancer cells was determined using the three-dimensional collagen matrix migration assay, as previously described [3, 53]. In brief, 50 µl of a cell suspension containing 4 × 10^4^ cells and supplements (100 ng/ml EGF, 100 nM 5-fluoro-2-indolyl-des-chlorohalopemide (FIPI) or a combination of both (all from Sigma-Aldrich, Taufkirchen, Germany)) was mixed with 100 µl of a collagen solution composed of liquid collagen (PureCol,CellSystems GmbH, Troisdorf, Germany), 10 × minimal essential medium (MEM) (Sigma-Aldrich, Taufkirchen, Germany) and 7.5% sodium bicarbonate solution (Sigma-Aldrich, Taufkirchen, Germany). Subsequently, the cell suspension-collagen solution mixture was filled in self-constructed migration chambers and incubated for 30–45 min at 37 °C to allow collagen to polymerize. Thereafter, the migration chambers were filled with media (and supplements), sealed at the fourth side, and placed on a heat plate (37 °C) under a microscope. Cell migration was recorded for at least 15 h by time-lapse video microscopy and was analyzed by computer-assisted cell tracking. Therefore, 30 cells were randomly chosen, and two-dimensional projections of the paths were digitized as x/y coordinates in 15 min intervals. A detailed protocol, including video tutorials of the preparation of migration chambers, cell tracking, and data analysis, is given in Rommerswinkel et al. [53].

### Flow cytometry-based measurements of changes in intracellular calcium levels

Changes in intracellular calcium levels of MDA-NEO and MDA-HER2 human breast cancer cells were determined using a FACSCalibur flow cytometer (Becton Dickenson, Heidelberg, Germany), as described previously by Gergely et al. [16], [3, 39, 55]. In brief, cells (5 × 10^5^) were stained with Fluo-4 (Thermo Fisher Scientific, Bonn, Germany) at 37 °C, according to the manufacturer’s instructions. Depending on the experiment, cells were pretreated for a defined time interval with the following inhibitors at 37 °C: U73122 (10 µM, 5 min), edelfosine (10 µM, 25 µM, 50 µM; 30 min), FIPI (1 nM, 10 nM, 100 nM; 1 min, 5 min, 10 min, 30 min), PRE084 (0.00025 µM, 0.0025 µM, 0.025 µM, 0.25 µM, 2.5 µM, 25 µM), BD1047 (0.00025 µM, 0.0025 µM, 0.025 µM, 0.25 µM, 2.5 µM, 25 µM) and PPBP maleate (0.00025 µM, 0.0025 µM, 0.025 µM, 0.25 µM, 2.5 µM, 25 µM, 250 µM). U73122, FIPI, edelfosine, PRE084, BD1047 and PPBP maleate were all purchased from Sigma-Aldrich, Taufkirchen, Germany. Acquisition was paused after 50 s for 15 s, whereby either 100 ng/ml EGF or PA (10 µM, 50 µM, 100 µM; Sigma-Aldrich, Taufkirchen, Germany) was added or the tube was left untreated for control purposes. PA solution was generated according to Bohdanowicz et al. [5], Schlam et al. [54]. Briefly, PA was first air-dried of chloroform and then dissolved in media containing 4 mg of fatty acid-free albumin (Sigma-Aldrich, Taufkirchen, Germany) to yield a 100 mM stock solution. The tube was mixed, and the acquisition was continued for a total of 204.80 s. Changes in intracellular calcium levels were quantified by determining the mean fluorescence intensity (MFI) at 10 s intervals using the WinMDI software application (Version 2.9; www.cyto.purdue.edu/flowcyt/software/Winmdi.htm). The mean MFI of intervals 2–4 (equal to 20–50 s) served as a baseline, which was set to 100%, whereas the mean MFI of the first 30 s after stimulation was defined as the rate of calcium release that was calculated in relation to the baseline level.

### siRNA mediated PLD1 knockdown

Knockdown of PLD1 expression was achieved by specific PLD1 small interfering RNA (siRNA; sc-44000; Santa Cruz Biotechnology, Heidelberg, Germany). Scrambled control RNA (scRNA; Qiagen GmbH, Hilden, Germany) was used for control experiments. MDA-NEO and MDA-HER2 cells (1 × 10^6^) were transfected with 100 nM siRNA and 100 nM scRNA, respectively, by electroporation using the Nucleofector™ 2b Device (Lonza, Cologne, Germany) as described in the user’s manual. Electroporated cells were seeded in T25 cell culture flasks (Sarstedt, Nümbrecht, Germany) for up to 72 h. Knockdown of PLD1 expression was verified by qPCR and PLD1 immunoprecipitation.

### qPCR

The siRNA-mediated knockdown of PLD1 expression in MDA-NEO and MDA-HER2 cells was determined by qPCR. Cells transfected with either scRNA- or PLD1-specific siRNA were harvested, and the total RNA was isolated using the NucleoSpin® RNA Kit (Macherey Nagel, Düren, Germany) in accordance with the instruction manual. The purity and concentration of isolated RNA were checked by UV photometry. The RevertAid First Strand cDNA Synthesis Kit (Thermo Fisher Scientific GmbH, Schwerte, Germany) was used for cDNA synthesis according to the provided protocol, and the cDNA concentration and purity were determined by UV photometry. qPCR analysis was performed by using the StepOne Plus Real-Time PCR System (Thermo Fisher Scientific GmbH, Schwerte, Germany), TaqMan® Fast Advanced Master Mix (Thermo Fisher Scientific GmbH, Schwerte, Germany) and specific TaqMan® probes for PLD1 (Hs01111354_m1; Thermo Fisher Scientific GmbH, Schwerte, Germany), PLD2 (Hs01093234_m1; Thermo Fisher Scientific GmbH, Schwerte, Germany) and GAPDH (Hs02758991_g1; Thermo Fisher Scientific GmbH, Schwerte, Germany) following the manufacturer’s instructions. The 2^−ΔCT^ method was used to determine the relative PLD1 and PLD2 expression levels in relation to the housekeeping gene GAPDH.

### Immunoprecipitation

To demonstrate siRNA-mediated PLD1 knockdown at the protein level, PLD1 was immunoprecipitated and subsequently determined by Western blot analysis. Briefly, siRNA- and scRNA-electroporated cells (1 × 10^6^) were cultured for 72 h in T25 cell culture flasks (Sarstedt, Nümbrecht, Germany). The medium was removed, and the cells were washed once with ice-cold PBS. Subsequently, 500 µl of ice-cold lysis buffer (Cell Signaling Technology Europe, Frankfurt am Main, Germany) supplemented with protease cocktail inhibitor (Cell Signaling Technology Europe, Frankfurt am Main, Germany) and 1 mM PMSF (Sigma Aldrich, Taufkirchen, Germany) was added, and the cells were incubated for 5 min on ice. Thereafter, the cells were scraped, transferred to a reaction tube, incubated for an additional 30 min on ice and then centrifuged (14.000 × g, 10 min, 4 °C). The supernatant was transferred into a new reaction tube, and the protein concentration was determined using the Pierce™ BCA Protein Assay Kit (Thermo Fisher Scientific, Schwerte, Germany) in accordance with the instruction manual. A total of 500 µg of protein was used for immunoprecipitation. Protein-A magnetic beads (25 µl; New England Biolabs GmbH, Frankfurt am Main, Germany) were prewashed two times with lysis buffer. Then, primary antibodies were added (anti-PLD1: 1:50; Cell Signaling, Frankfurt am Main, Germany; rabbit IgG: 2.5 µg: Santa Cruz Biotechnology Inc., Heidelberg, Germany; Table 1) and incubated overnight at 4 °C with mild rotation. PLD1 and IgG-bound Protein A magnetic beads were washed two times with lysis buffer. Prior to immunoprecipitation, cell lysates were preincubated with Protein A magnetic beads for 20 min under mild rotation to remove proteins that could bind nonspecifically to Protein A. Subsequently, such cell lysates were incubated with PLD1 and IgG-bound Protein A magnetic beads for 1 h at 4 °C with mild rotation. Finally, samples were washed three times with lysis buffer, resuspended in 3 × Laemmli buffer (with DTT, without β-mercaptoethanol) and incubated for 3 min at 95 °C. Beads were removed by placing the reaction tubes in a magnetic rack. Immunoprecipitated PLD1 was detected by Western blot analysis, whereby a different PLD1 was used (PLD1, rabbit monoclonal, clone EP1506Y; Abcam, Berlin, Germany; Table 1).

**Table 1:** Summary of antibodies used in this study

Antibodies	Purchase order number	Supplier
EGFR, rabbit mAb, clone C74B9	#2646	CST^a^
Phospho-EGFR Tyr992, rabbit polyclonal	#2235	CST^a^
HER2/ErbB2, rabbit mAB, clone 29D8	#2165	CST^a^
Phospho-HER2/ErbB2 Tyr1196, rabbit mAb, clone D66B7	#6942	CST^a^
AKT (pan), rabbit mAb, clone 11E7	#4685	CST^a^
Phospho-AKT Ser473, rabbit mAb, clone D9E	#4060	CST^a^
p44/42 MAPK (ERK1/2), rabbit polyclonal	#9102	CST^a^
Phospho-p44/42 (ERK1/2) Thr202/Tyr204, rabbit polyclonal	#9101	CST^a^
PLC-γ1, rabbit mAb, clone D9H10	#5690	CST^a^
Phospho-PLC-γ1 Tyr783, rabbit mAb, clone D6M9S	#14008	CST^a^
PLD1, rabbit polyclonal	#3832	CST^a^
PLD1, rabbit monoclonal, clone EP1506Y	ab68150	Abcam^b^
Sigma-1-receptor, mouse mAb, clone B-5	sc-137075	Santa Cruz^c^
elf4E, rabbit polyclonal	#9742	CST^a^
β-actin, rabbit mAb, clone 13E5	#4970	CST^a^
rabbit IgG	sc3888	Santa Cruz^c^
Anti-mouse-IgG-HRP-linked	#7076	CST^a^
Anti-rabbit-IgG-HRP-linked	#7074	CST^a^

^a^Cell Signaling Technology Europe, Frankfurt am Main, Germany

^b^Abcam, Berlin, Germany

^c^Santa Cruz Biotechnology Inc., Heidelberg, Germany

### Western blot analysis

MDA-NEO and MDA-HER2 cells (2 × 10^5^/20 µl) were treated with EGF (100 ng/ml; Sigma-Aldrich, Taufkirchen, Germany) and/or FIPI (100 nM) at 37 °C using the following conditions: control (untreated), 5 min EGF, 1 min FIPI, 1 min FIPI + 5 min EGF, 5 min FIPI, 5 min FIPI + 5 min EGF, 10 min FIPI, 10 min FIPI + 5 min EGF, 30 min FIPI, and 30 min FIPI + 5 min EGF. Subsequently, 10 µl of 3 × Laemmli buffer was added, and samples were lysed for 10 min at 95 °C. Proteins were separated by 8% or 10% sodium dodecyl sulfate–polyacrylamide gel electrophoresis (SDS-PAGE) and subsequently transferred to polyvinylidene difluoride (PVDF) membranes (Merck Millipore, Darmstadt, Germany) under semi-dry conditions. Membranes were either blocked with 10% (w/v) nonfat milk powder (Applichem, Darmstadt, Germany) or 5% BSA (Sigma-Aldrich, Taufkirchen, Germany) in TBS-T. Bands were visualized by using the Pierce ECL Western blot substrate (Thermo Fisher Scientific, Bonn, Germany) as recommended by the manufacturers’ instructions and the Aequoria Macroscopic Imaging System (Hamamatsu Photonics Germany, Herrsching am Ammersee, Germany). The primary and secondary antibodies used in this study are summarized in Table 1. ImageJ (imagej.nih.gov/ij/) was used for densitometric analysis of the Western blots.

### Statistical analysis

The statistical significance of the data was determined using GraphPad PRISM software (version 8.3.1). All results are presented as the mean ± S.E.M. from at least three independent experiments. Normally distributed data were analyzed by ordinary one-way ANOVA followed by Tukey’s test, whereas the Kruskal–Wallis test followed by Dunn’s multiple correction test was used for nonnormally distributed data. A p-value of < 0.05 was considered significant.

## Results

### The migratory activities of MDA-NEO and MDA-HER2 breast cancer cells were significantly impaired by the PLD inhibitor FIPI

**Fig. 1:**
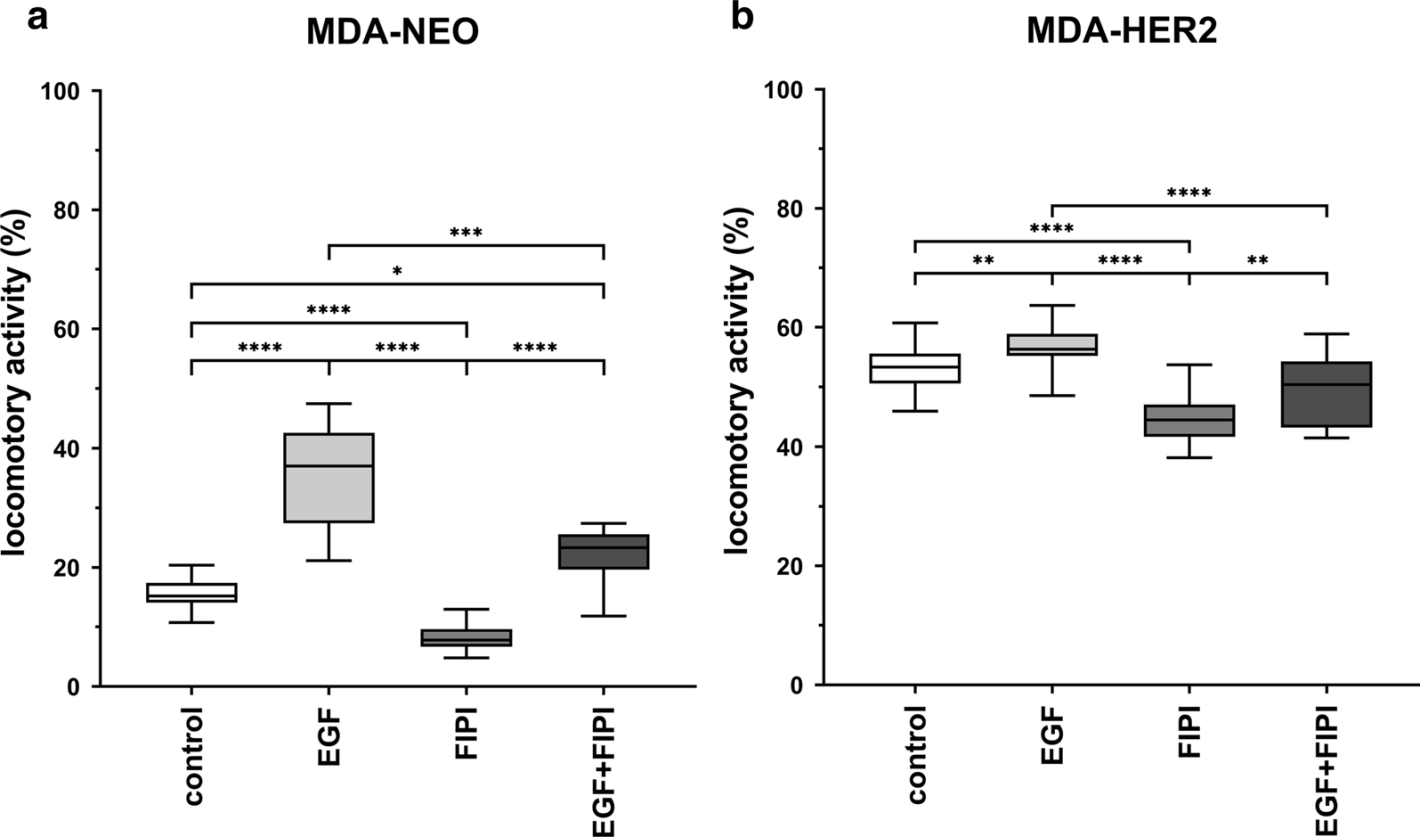
The EGF-induced migration of MDA-NEO and MDA-HER2 breast cancer cells is significantly impaired by the PLD inhibitor FIPI. MDA-NEO cells (**a**) and MDA-HER2 cells (**b**) were treated with 100 ng/ml EGF, 100 nM FIPI and a combination of both. The results are presented as the mean of three independent experiments. Statistical analysis: one-way ANOVA and Kruskal–Wallis post hoc test: * = *p* < 0.05, ** = *p* < 0.01, *** = *p* < 0.001, **** = *p* < 0.0001

To investigate the impact of PLD activity on the migration of MDA-NEO and MDA-HER2 human breast cancer cells, the locomotory activity of the cells was investigated using the specific PLD inhibitor FIPI [58]. MDA-NEO cells are EGFR positive, whereas MDA-HER2 cells express both EGFR and HER2 [12, 52, 53]. Both the spontaneous and 100 ng/ml EGF-induced migration of MDA-NEO and MDA-HER2 human breast cancer cells was significantly blocked by 100 nM FIPI (Fig. 1). In accordance with previous studies [52, 53], the locomotory activity of MDA-NEO cells was significantly increased by EGF (control: 15.2% (median) vs. 100 ng/ml EGF: 35.1% (median); *p* < 0.0001; Fig. 1a). Treatment with 100 nM FIPI significantly impaired the migratory activity of untreated and EGF-stimulated MDA-NEO cells (100 nM FIPI: 7.8% (median), 100 nM FIPI + 100 ng/ml EGF: 23.3% (median); Fig. 1a). Similar data were obtained for MDA-HER2 human breast cancer cells. A slight but significant EGF-induced migration of the cells was observed that was significantly impaired by FIPI (median values: control: 53.3%, 100 ng/ml EGF: 56.3% (*p* < 0.01 vs. control), 100 nM FIPI: 44.4% (*p* < 0.001 vs. control) and 100 nM FIPI + 100 ng/ml EGF: 50.4% (*p* < 0.0001 vs. 100 ng/ml EGF); Fig. 1b).

### The PLD inhibitor FIPI potently blocks EGF-induced calcium release in MDA-NEO and MDA-HER2 human breast cancer cells

**Fig. 2:**
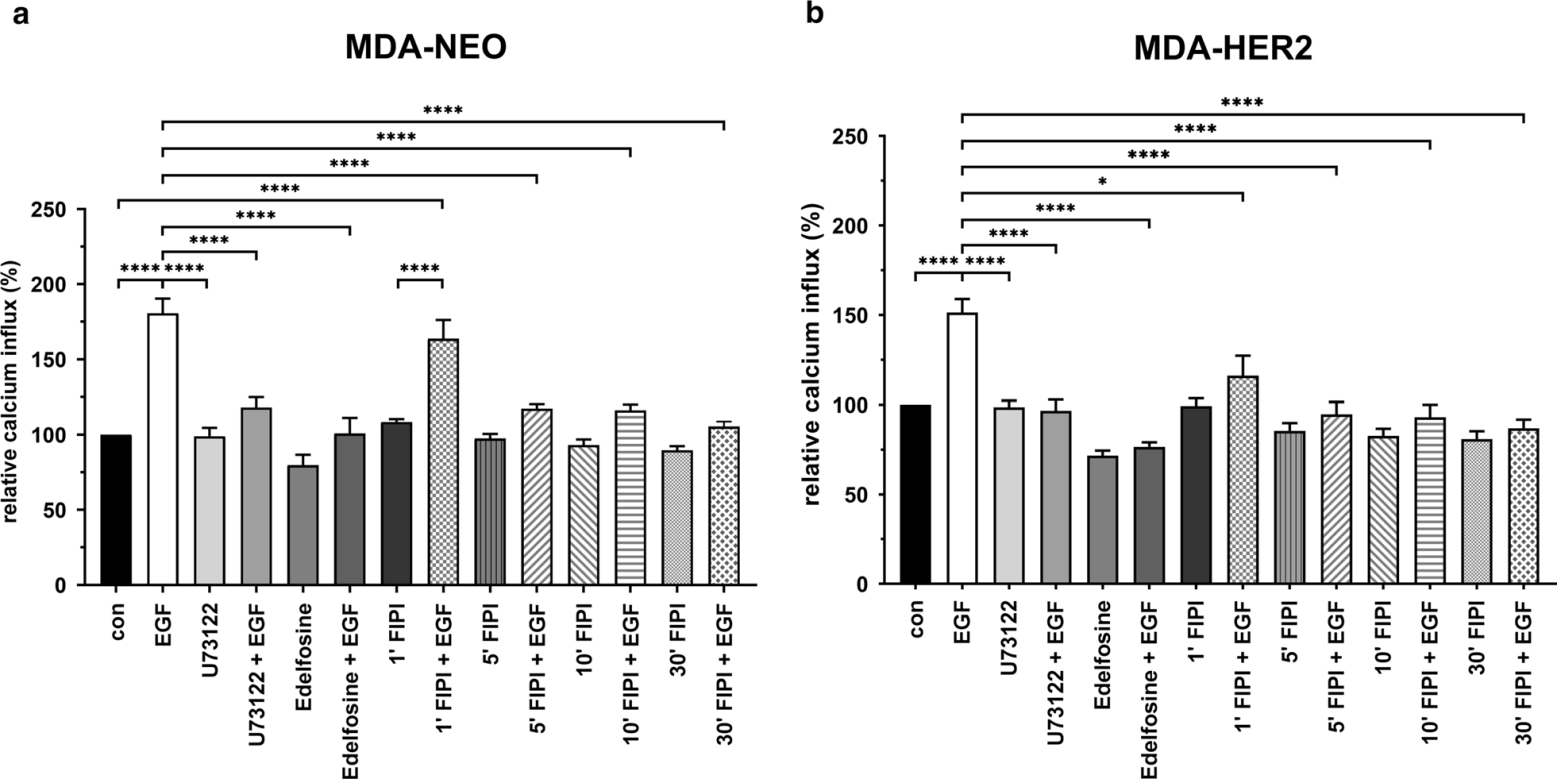
EGF-induced calcium release in MDA-NEO and MDA-HER2 breast cancer cells is significantly blocked by the PLD inhibitor FIPI. MDA-NEO cells (**a**) and MDA-HER2 cells (**b**) were treated with 100 ng/ml EGF, 10 µM U73122, 10 µM edelfosine and appropriate combinations. For FIPI experiments, cells were pretreated with 100 nM FIPI as indicated. The results are presented as the mean ± S.E.M. of at least three to four independent experiments. Statistical analysis: one-way ANOVA and Tukey’s post hoc test: * = *p* < 0.05, ** = *p* < 0.01, *** = *p* < 0.001, **** = *p* < 0.0001

It is well recognized that PLC-γ1 activity concomitant with the release of calcium from intracellular stores plays a crucial role in cell migration [48, 50]. Since PLC-γ1 could be activated in a RTK-dependent and RTK-independent manner, we wondered about the impact of PLD in this process since Jones and Carpenter showed that the PLD product PA appears to additionally activate PLC-γ1 by acting as an allosteric modifier [27]. Hence, calcium measurements with EGF, U73122, and FIPI were performed with both breast cancer cell lines. As expected, EGF-induced calcium release from intracellular stores was observed in both cell lines and was significantly blocked by the PLC-γ1 inhibitors U73122 (10 µM) and edelfosine (10 µM,Fig. 2, Additional file 2: Fig. S1). Interestingly, FIPI (100 nM) blocked EGF-induced calcium release in both breast cancer cell lines, whereby a significant inhibition was already observed in MDA-HER2 cells after 1 min of FIPI preincubation (Fig. 2b). In contrast, 5 min of preincubation with FIPI was mandatory for significantly inhibiting EGF-induced calcium release in MDA-NEO breast cancer cells (Fig. 2a). Interestingly, slight EGF-induced calcium release was observed in all FIPI-pretreated cells in comparison to appropriate FIPI controls (Fig. 2a, b). In addition to 100 nM FIPI, different concentrations were tested and revealed that even 30 min of preincubation with 1 nM FIPI sufficiently abrogated EGF-induced calcium release in both MDA-NEO and MDA-HER2 breast cancer cells (Additional file 2: Fig. S1). We also tested whether PA (10 µM, 50 µM, and 100 µM) was able to induce the release of calcium from intracellular stores via direct PLC-γ1 activation. Indeed, very weak enhancement of intracellular calcium was observed in both cell lines stimulated with different PA concentrations (Additional file 2: Fig. S1), but it is not yet clear whether this was attributed to a real PA effect.

### The pretreatment of MDA-NEO and MDA-HER2 cells with FIPI resulted in slightly altered EGFR, HER2, and ERK1/2 phosphorylation levels

**Fig. 3:**
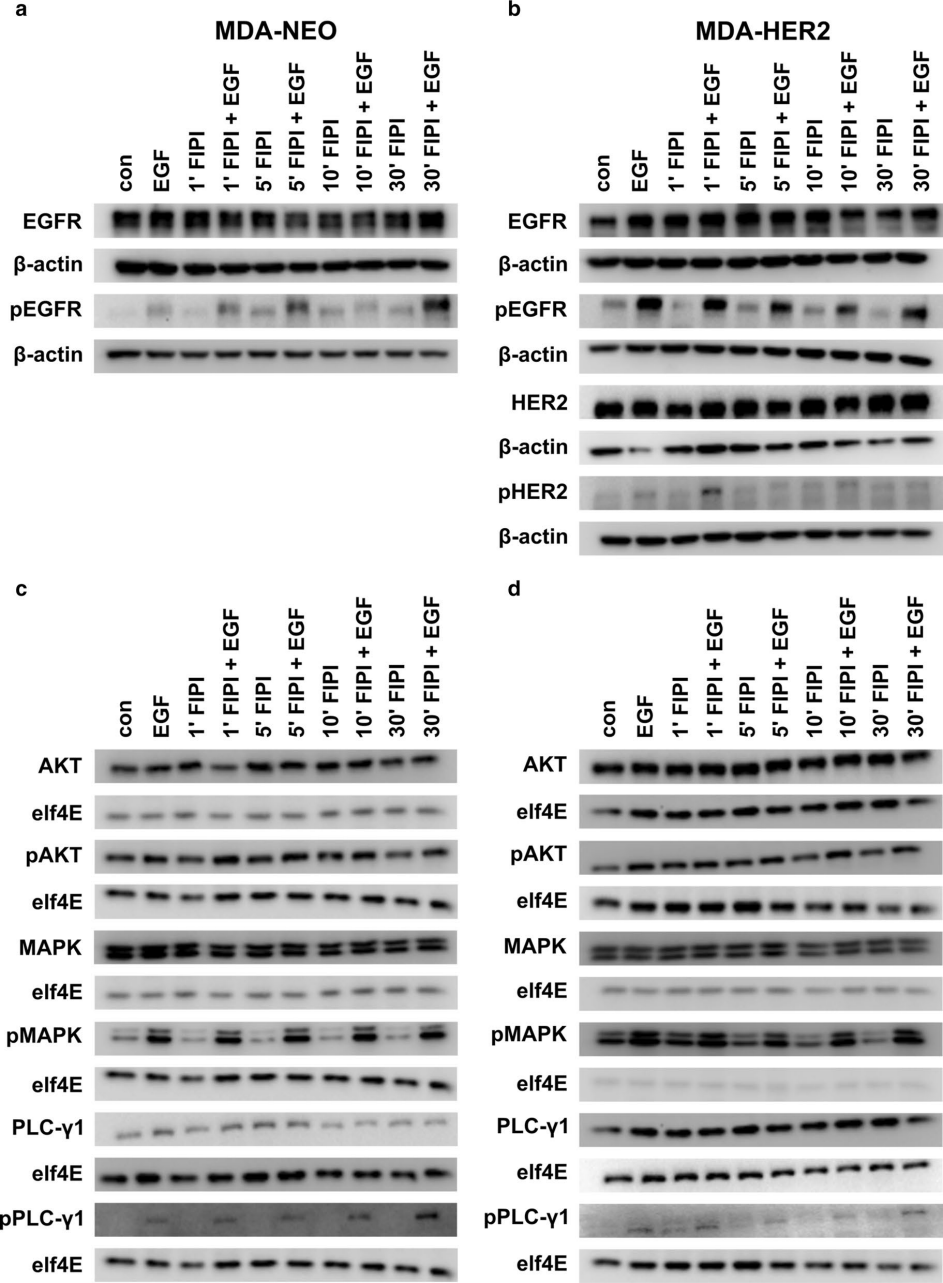
FIPI has no impact on the expression and phosphorylation levels of RTKs and signal transduction molecules. MDA-NEO cells (**a**, **c**) and MDA-HER2 cells (**b**, **d**) were pretreated with 100 nM FIPI as indicated and then stimulated with 100 ng/ml EGF. Representative Western blot data from at least four independent experiments are shown

FIPI is a well-known PLD inhibitor [58], but it has not yet been associated with inhibiting PLC-γ1-mediated calcium release in cells. To explore how FIPI interferes in this process, we first analyzed the overall expression levels and phosphorylation patterns of EGFR, HER2, AKT, MAPK^p42/44^, and PLC-γ1 in both cell lines in the presence of EGF and FIPI. AKT, MAPK^p42/44^ and PLC-γ1 were chosen to determine the activities of RAS-RAF-MAPK, PI3K-AKT, and PLC-γ1 signaling. Briefly, the pretreatment of cells with 100 nM FIPI for up to 30 min showed no alterations in target protein expression levels in either cell line (Fig. 3; Additional files 3, 4, 5: Figs. S2, S3, S4). In contrast, basal and EGF-induced pEGFR levels were significantly and time-dependently increased in FIPI-treated MDA-NEO cells (Fig. 3a; Additional file 3: Fig. S2). Interestingly, this reproducible effect was only observed in the MDA-NEO breast cancer cell line but not in MDA-HER2 cells. Here, pEGFR (and pHER2) levels remained unaltered in FIPI- and EGF + FIPI-treated cells compared to appropriate controls (Fig. 3b; Additional file 3: Fig. S2).

In accordance with the higher pEGFR levels in EGF and FIPI cotreated MDA-NEO cells, we observed slightly increased pPLC-γ1 levels in this cell line (Fig. 3c; Additional file 5: Fig. S4), whereas pAKT and pMAPK^p42/44^ levels in EGF + FIPI treated MDA-NEO cells were comparable to those of the EGF control. However, prolonged FIPI incubation was correlated with slightly diminished basal pMAPK^p42/44^ levels in MDA-NEO breast cancer cells (Fig. 3c; Additional file 4: Fig. S3), which was also observed in MDA-HER2 cells (Fig. 3d; Additional file 4: Fig. S3). In summary, these findings indicate that the phosphorylation pattern of signal transduction molecules in MDA-NEO and MDA-HER2 cells could be altered by prolonged treatment with FIPI.

### The siRNA-mediated knockdown of PLD1 significantly impairs EGF-induced calcium release in MDA-NEO and MDA-HER2 cells

**Fig. 4:**
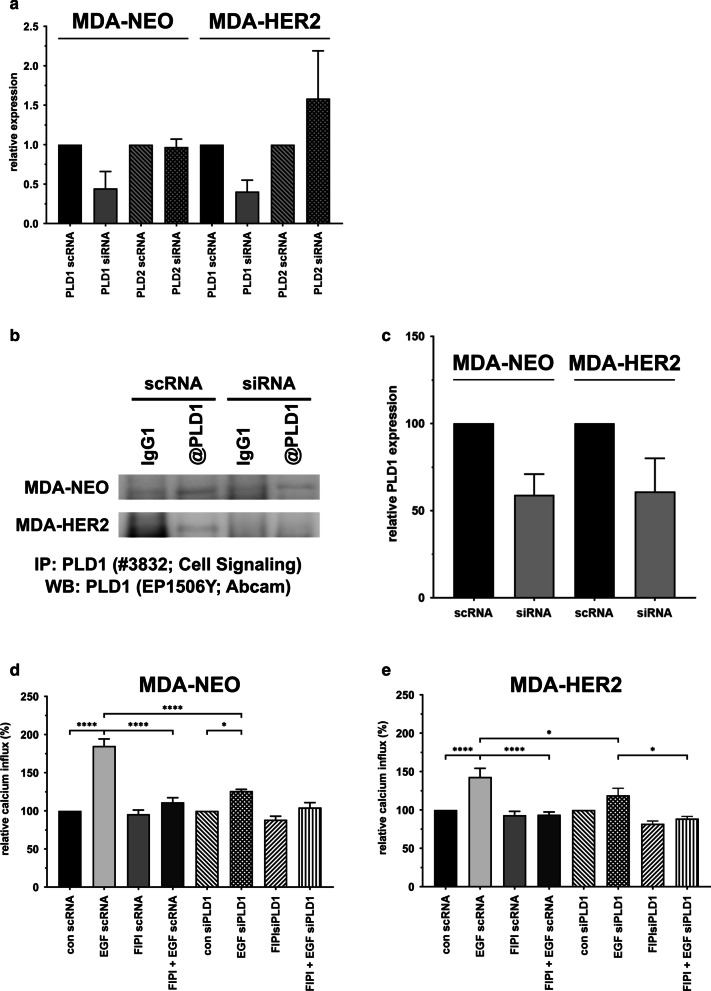
EGF-induced calcium is significantly blocked after PLD1 knockdown. (**a**) qPCR data of relative PLD1 and PLD2 expression in PLD1 siRNA- and scRNA-transfected MDA-NEO and MDA-HER2 cells. The results are presented as the mean of two independent experiments. (**b**) Western blot data of immunoprecipitated PLD1 in scRNA- and siRNA-transfected MDA-NEO and MDA-HER2 cells. Representative blots of two independent experiments are shown. (**c**) Densitometric analysis of PLD1 IP/WB data. The results are presented as the mean ± S.E.M. of two independent experiments. Transfected MDA-NEO cells (**d**) and MDA-HER2 cells (**e**) were pretreated for 30 min with 100 nM FIPI and then stimulated with 100 ng/ml EGF. The results are presented as the mean ± S.E.M. of at least four independent experiments. Statistical analysis: one-way ANOVA and Tukey’s post hoc test: * = *p* < 0.05, ** = *p* < 0.01, *** = *p* < 0.001, **** = *p* < 0.0001

Since elevated pEGFR levels were observed in EGF + FIPI-cotreated MDA-NEO cells or remained unaltered in MDA-HER2 cells, we concluded that FIPI-impaired EGF-induced calcium release was not attributed to differential receptor phosphorylation kinetics. To investigate whether the inhibition of PLD activity correlated with impaired EGF-mediated calcium release, first, the relative expression levels of PLD1 and PLD2 in MDA-NEO and MDA-HER2 cells were determined by conventional and qPCR. The results showed that PLD1 was predominantly expressed in both cell lines (data not shown). The transfection of cells with specific PLD1 siRNA revealed that PLD1 expression was markedly impaired after 72 h (Fig. 4a). In contrast, PLD2 expression levels remained unaltered in comparison to scRNA controls (Fig. 4a), indicating that the siRNA used was PLD1-specific. Next, calcium measurements were performed, which clearly showed that EGF-induced calcium release was significantly blocked in PLD1 siRNA-treated MDA-NEO and MDA-HER2 cells compared to appropriate scRNA controls. Hence, PLD1 activity seems necessary for EGF-induced calcium release in MDA-NEO and MDA-HER2 human breast cancer cells.

### The inhibition of the sigma-1 receptor abrogates EGF-induced calcium release in MDA-NEO and MDA-HER2 cells

**Fig. 5:**
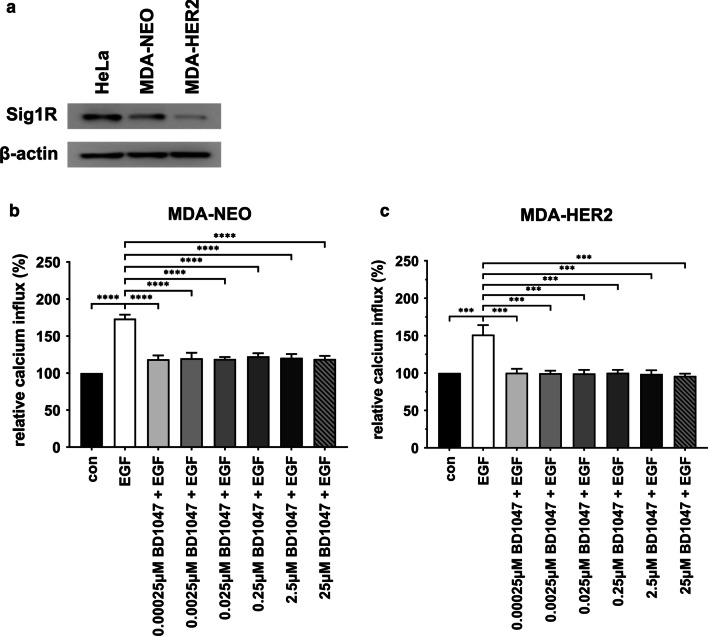
Inhibition of Sig-1R also abrogates EGF-induced calcium release in MDA-NEO and MDA-HER2 cells. **a** MDA-NEO and MDA-HER2 breast cancer cells express Sig-1R. HeLa served as a positive control. Representative Western blot data from three independent experiments are shown. MDA-NEO cells (**b**) and MDA-HER2 cells (**c**) were pretreated with different concentrations of the PLD1 antagonist BD1047 and then stimulated with 100 ng/ml EGF. The results are presented as the mean ± S.E.M. of three independent experiments. Statistical analysis: one-way ANOVA and Tukey’s post hoc test: * = *p* < 0.05, ** = *p* < 0.01, *** = *p* < 0.001, **** = *p* < 0.0001

To further clarify the role of PLD1 activity in EGF-induced calcium release, we next focused on the sigma-1 receptor (Sig-1R), which is an integral ER membrane protein [7]. Sig-1R binds diverse ligands and regulates many signaling proteins, including IP3R, that release calcium from the ER upon IP3-mediated activation [7]. Likewise, it was demonstrated that Sig-1R drives breast and colorectal cancer cell migration by triggering cellular calcium homeostasis [20]. Western blot data showed that both MDA-NEO and MDA-HER2 breast cancer cells were positive for Sig-1R expression, whereby higher Sig-1R protein levels were observed in MDA-NEO cells than in MDA-HER2 cells (Fig. 5a). To study the impact of Sig-1R activity on EGF-induced calcium signaling, MDA-NEO and MDA-HER2 cells were preincubated with different concentrations of the specific Sig-1R antagonist BD1047 [7]. The results showed that BD1047 potently blocked EGF-induced calcium release in both cell lines, whereby even relatively low BD1047 concentrations of approximately 0.00025 µM were capable of impairing calcium signaling in the cells (Fig. 5b, c). Briefly, these data likely indicate that Sig-1R might be involved in the EGF-induced calcium release of MDA-NEO and MDA-HER2 breast cancer cells.

### The activation of the sigma-1 receptor also abrogates EGF-induced calcium release in MDA-NEO and MDA-HER2 cells

**Fig. 6:**
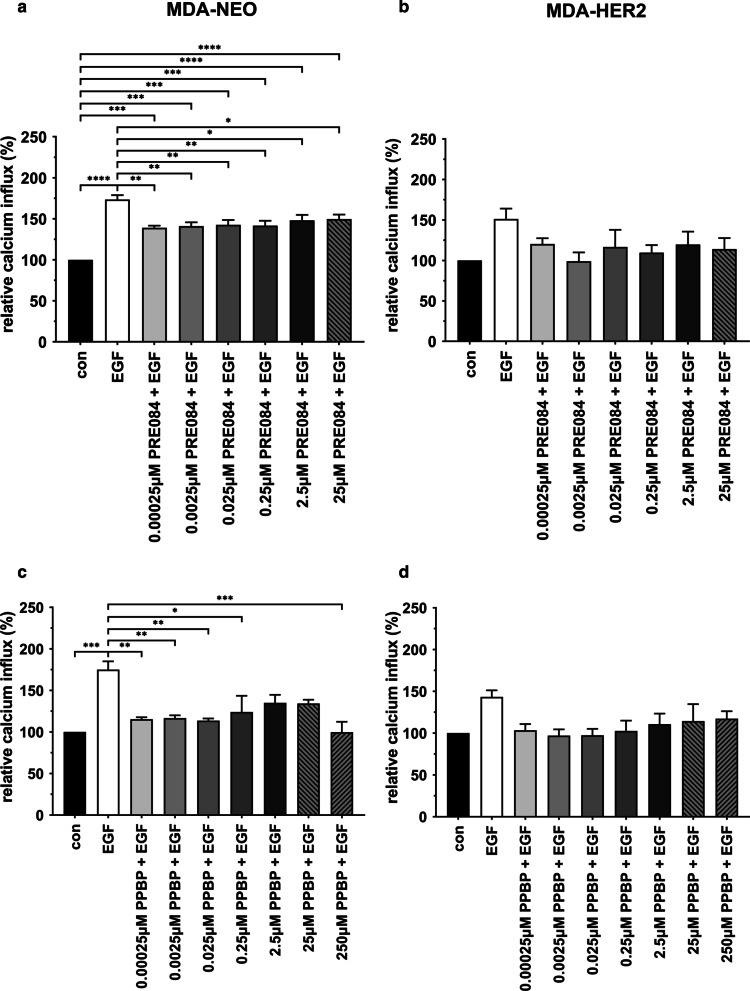
EGF-induced calcium release in MDA-NEO and MDA-HER2 cells is also impaired by PLD1 agonists. MDA-NEO cells and MDA-HER2 cells were pretreated with different concentrations of the PLD1 agonist PRE084 (**a**: MDA-NEO; **b**: MDA-HER2) or PPBP maleate (**c**: MDA-NEO; **d**: MDA-HER2) and then stimulated with 100 ng/ml EGF. The results are presented as the mean ± S.E.M. of three independent experiments. Statistical analysis: **a**–**c** One-way ANOVA and Tukey’s post hoc test: * = *p* < 0.05, ** = *p* < 0.01, *** = *p* < 0.001, **** = *p* < 0.0001. **d** One way ANOVA and Kruskal–Wallis post hoc test

To further conclude that Sig-1R activity plays a role in EGF-induced calcium release in MDA-NEO and MDA-HER2 breast cancer cells, further measurements were performed by using the specific Sig-1R agonist PRE084. It was expected that comparable or even higher EGF-mediated intracellular calcium concentrations would be observed in both cell lines after preincubation with PRE084. Interestingly, pretreatment with PRE084 resulted in slightly decreased EGF-induced intracellular calcium levels in both cell lines (Fig. 6a, b). To prove this observation, similar experiments have been extended with another Sig-1R agonist, PPBP maleate. According to the PRE084 data (Fig. 6a, b), the preincubation of cells with PPBP maleate also resulted in impaired EGF-induced intracellular calcium levels (Fig. 6c, d).

## Discussion

In the present study, we investigated the role of PLD in the EGF-induced migration of MDA-NEO and MDA-HER2 breast cancer cells. Briefly, our data show that the treatment of both cell lines with the specific PLD inhibitor FIPI significantly impairs the spontaneous and matrix-induced migration of both cell lines. This demonstrates, consistent with other studies, that the knockdown of PLD1 expression or blocking of PLD activity with specific inhibitors correlates with impaired (cancer) cell migration [11, 22, 30, 36, 41, 43, 58]. PLD is involved in different pathways that lead to the reorganization of the actin cytoskeleton and induction of cell migration and chemotaxis. Knapek and colleagues proposed that the participation of PLD in chemotaxis and migration of macrophages and fibroblasts involves three major pathways depending on either protein–protein interactions or enzymatic activity [36]. Therefore, actin polymerization was attributed to PLD-derived PA-induced mTOR and S6K signaling, the formation of a PLD2-Grb2 protein complex and downstream activation of MAPK signaling or a direct (or indirect) interaction of PLD and PA (and WASP) with actin [36]. Similar findings were presented by Henkels et al., showing that the formation of the PLD2-Grb2 protein complex resulted in Rac2 activation and actin polymerization in MDA-MB-231 breast cancer cells [22]. Likewise, PLD2-derived PA directly or indirectly induced actin polymerization via WASP (which may also bind to the PLD2-Grb2 complex) [22]. Lingrand and coworkers recently showed that high expression levels of stearoyl-CoA desaturase-1 (SCD1) promoted the migration of MDA-MB-231 breast cancer cells via the formation of oleic acid and the subsequent induction of PLD-mTOR-S6K signaling [41]. The knockdown of PLD2 expression, but not PLD1, by siRNA correlated with diminished phosphorylated S6K levels in MDA-MB-468 cells, suggesting that PLD2 rather than PLD1 was likely mandatory for mTOR and S6K signaling [34]. Which of these PLD-dependent pathways plays a role in the EGF-induced migration of MDA-NEO and MDA-HER2 breast cancer cells remains unclear. Given that PLD2 is mandatory for mTOR and S6K signaling in MDA-MB-468 breast cancer cells [34], it can be concluded that this pathway was most likely involved in MDA-NEO and MDA-HER2 cell migration since both cell lines were derived from MDA-MB-468 cells [9, 12]. However, it cannot be ruled out that other PLD-dependent pathways, such as the PLD2-Grb2 complex or MAPK signaling, might also be involved in the migration of MDA-NEO and MDA-HER2 breast cancer cells.

A rather unexpected and not yet fully understood finding of this study was that the PLD inhibitor FIPI potently blocked EGF-induced calcium release from intracellular stores in both cell lines. Since FIPI data could be validated with siRNA-mediated PLD1 knockout in both cell lines, we concluded that the observed effects were most likely attributed to the PLD1 isoform. Likewise, qPCR data revealed slightly higher PLD1 than PLD2 expression levels in MDA-NEO and MDA-HER2 cells, which is in agreement with data of Kim et al. showing a similar PLD1/PLD2 expression profile in MDA-MB-468 wild type, from which MDA-NEO and MDA-HER2 cells were derived. Calcium is a universal second messenger that is involved in various cellular processes, including proliferation, apoptosis, transcription, metabolism and cell migration [6, 13, 19]. In this regard, we and others have already demonstrated the crucial interplay of RTK-mediated PLC-γ1 activation, the release of calcium from intracellular stores, the reorganization of the actin cytoskeleton and cell migration [3, 12, 32, 40, 48, 52, 56, 62, 65]. Likewise, it is well known that the inhibition of the RTK-PLC-γ1 interplay abolishes the release of calcium from intracellular stores and cell migration [3, 12, 32, 40, 48, 52, 56, 62, 65]. Hence, we conclude from our data that either FIPI itself or the inhibition of PLD1 activity interferes with this interplay.

Data from Hatton and colleagues indicated that EGFR expression levels in human breast cancer cells were related to PLD2 expression and its enzymatic reaction product PA [21]. Therefore, increased PLD2 expression and PA levels in MDA-MB-231 breast cancer cells were directly correlated with higher EGFR expression levels [21]. Likewise, the knockdown of PLD2 expression or inhibition of PLD2 activity by FIPI resulted in lower EGFR expression levels in the cells [21]. However, MDA-MB-231 breast cancer cells in the work of Hatton et al. were treated for up to 48 h with 200 nM and 700 nM FIPI [21], which was much higher and longer than in this study. Here, a 30 min preincubation with 1–100 nM FIPI significantly impaired EGF-induced calcium release in both cell lines (Additional file 2: Fig. S1). Moreover, similar results were achieved with 100 nM FIPI and 5 min preincubation (Fig. 2), and Western blot data further revealed that EGFR expression levels remained unaltered in FIPI-pretreated cells (Fig. 3), indicating that short-term exposure to FIPI did not alter cell EGFR levels. Hence, the possibility that the impaired EGF-induced calcium release was attributed to FIPI-mediated lower EGFR expression levels [21] can be ruled out. However, with regard to cell migration data, we cannot exclude that time-dependent FIPI-mediated EGFR down-regulation might have occurred since MDA-NEO and MDA-HER2 cells were treated with 100 nM FIPI for up to 15 h in the cell migration experiments.

In accordance with our findings, Bates and colleagues also observed that FIPI could block the release of calcium from intracellular stores [4]. Studies on *Xenopus laevis* eggs revealed that PLD-derived PA led to Src stimulation, which subsequently activated PLC-γ1 concomitant with calcium release [4]. FIPI inhibited both PA production and Src phosphorylation in *Xenopus laevis* eggs concomitant with reduced calcium release [4] Likewise, PA addition to *Xenopus laevis* eggs was associated with Src and PLC-γ1 tyrosine phosphorylation, elevated IP3 levels and increased intracellular calcium levels [4]. Although the findings of Bates et al. nicely fit our data, it is clear that PLC-γ1 is predominantly activated by RTKs in many cell types [44, 49, 50, 59, 65], particularly by EGFR homodimers and EGFR/HER2 heterodimers in MDA-NEO and MDA-HER2 breast cancer cells [3, 9, 12, 32, 52]. Moreover, Bates et al. demonstrated that the inhibition of PA production correlated with decreased PLC-γ1 phosphorylation levels [4], which is opposite to our findings showing comparable pPLC-γ1 levels in EGF- and EGF + FIPI-treated MDA-NEO and MDA-HER2 cells. Hence, we conclude that the mechanism by which FIPI impairs EGF-induced calcium release in MDA-NEO and MDA-HER2 cells is different from the mechanism proposed by Bates and colleagues in *Xenopus laevis* eggs.

Jones and Carpenter demonstrated that PLC-γ1 activity also depends on PA [27]. Therefore, kinetic studies revealed that the activities of tyrosine-phosphorylated and control PLC-γ1 were increased 6- and 40-fold, respectively, by PA [27]. Given that PLD-derived PA plays a role in the PLC-γ1 activation state, it might be speculated that lower PA levels due to PLD inhibition by FIPI would be associated with decreased PLC-γ1 activity. Whether this possibility might be true remains to be elucidated. However, siRNA-treated MDA-NEO and MDA-HER2 cells showed decreased EGF-induced calcium release in FACS analysis but still expressed PLD2 (Fig. 4), which may also point to a PA-independent mechanism.

The stimulation of cells with EGF resulted in tyrosine-phosphorylated activated PLC-γ1 enzymes, even in the absence of PA [27]. Western blot studies clearly showed comparable pPLC-γ1 levels in EGF- and EGF + FIPI-treated MDA-NEO and MDA-HER2 cells, indicating that the tyrosine phosphorylation-dependent activation of PLC-γ1 was not affected by FIPI and active PLC-γ1 enzymes were present in the cells. Hence, the inhibition of PLD activity by FIPI or by siRNA-mediated PLD1 knockdown should have resulted in partial but not complete inhibition of EGF-induced calcium release. Interestingly, slightly increased intracellular calcium levels were observed in FIPI + EGF-treated cells compared to the appropriate FIPI controls, which might be attributed to only tyrosine-phosphorylated activated PLC-γ1 enzymes. This, however, has to be validated in ongoing studies.

Brailoiu and colleagues showed in neuroblastoma cells that IP3-evoked calcium signals could be additionally triggered through the choline-induced activation of sigma-1 receptors (Sig-1R), which increased the activity of IP3-stimulated IP3Rs [7]. Moreover, markedly elevated calcium signals were also detected in IP3- and choline-cotreated MCF-7 breast cancer cells that were stably transfected with a Sig-1R expression vector but not in MCF-7 wild-type cells [7]. Likewise, a constitutive enhancement of bradykinin-induced calcium release was observed in Sig-1R-expressing MCF-7 cells in comparison to nontransfected cells [64], indicating that the synergistic interaction between Sig-1R and IP3R and the release of calcium also works in breast cancer cells. In this regard, it was shown that some normal and most neoplastic breast epithelial cells and cell lines commonly expressed Sig-1R and that a high concentration of the nonspecific Sig-1R ligand haloperidol inhibited the growth of these cells and potentiated the effect of chemotherapy in vitro [61]. Moreover, data from Aydar et al. suggested a putative correlation between Sig-1R expression levels and the aggressiveness of breast cancer cell lines [2]. In any case, it is well known that the PLD-dependent cleavage of phosphatidylcholine results in the production of PA and choline. Hence, FIPI-mediated inhibition of choline production might be another mechanism by which this PLD inhibitor might impair EGF-induced calcium release in MDA-NEO and MDA-HER2 breast cancer cells. This assumption would be supported by Western blot data demonstrating Sig-1R expression in both breast cancer cell lines. Likewise, the specific Sig-1R inhibitor BD1047 significantly blocked EGF-induced calcium release in MDA-NEO and MDA-HER2 cells. However, Brailoiu and colleagues showed that choline alone was not capable of causing calcium release in Sig-1R-expressing MCF-7 breast cancer cells [7]. Only when IP3R was stimulated by IP3 was an additive choline-dependent Sig-1R effect observed [7]. Hence, the inhibition of choline production should have rather resulted in the blocking of this additive effect concomitant with partially impaired EGF-induced calcium release in both cell lines. Likewise, experiments with two different Sig-1R agonists (PRE084 and PPBP) also resulted in impaired EGF-induced calcium in both cell lines, which was rather unexpected and opposite to the data of Brailoiu et al., demonstrating that bradykinin-evoked calcium signals were enhanced by preincubation with the Sig-1R agonist PRE084. In contrast, data from Zhang and colleagues revealed that Sig-1R agonists, such as haloperidol, inhibited calcium channels in rat neurons from parasympathetic intracardiac ganglia and sympathetic superior cervical ganglia [67]. Here, the authors assumed that Sig-1R might alter the biophysical properties of calcium channels and attenuate whole calcium channel currents [67]. Whether a similar mechanism was responsible for the observed inhibition of EGF-induced calcium release in PRE084- and PPBP-treated MDA-NEO and MDA-HER2 cells remains unclear. However, it should be noted that both substances have not yet been tested in this context. Likewise, it is also not yet clear whether “classical” Sig1R agonists and antagonists do exist since several fundamental questions regarding the drug mechanism of action and the physiological relevance of aberrant Sig1R transcript and Sig1R protein expression in certain cancers remain unanswered or only partially answered [33].

## Conclusions

In the present study, we showed that the PLD inhibitor FIPI potently blocked EGF-induced calcium release and EGF-induced migration in MDA-NEO and MDA-HER2 breast cancer cells. However, the mode of action by which FIPI inhibition interferes with EGF-induced calcium release is not yet clear. Preincubation with FIPI for 5 min was sufficient to completely abolish EGF-induced calcium release in both cell lines, indicating a relatively fast mechanism. Likewise, tyrosine-phosphorylated PLC-γ1 levels remained unaltered in FIPI-treated cells, indicating that FIPI did not interfere directly with PLC-γ1 activation. Since the PLD product PA plays an important role in full PLC-γ1 activation [27], we conclude that the FIPI-mediated inhibition of EGF-induced calcium release was most likely attributed to lower PA levels due to the PLD blockade. It is further possible that Sig-1Rs were not activated due to lower choline levels [7] in FIPI-treated cells, which could also have contributed to decreased calcium release. If so, this would mean that both PA and choline would play a role in the EGF-induced and PLC-γ1-dependent release of calcium from intracellular stores. Calcium is a versatile second messenger that is involved in various cellular processes, including cell migration [6, 13, 19]. Hence, the abrogation of calcium signaling by FIPI is one explanation for the observed reduced migration of the cells in the presence of this inhibitor. However, PLD activity and the generation of PA are also mandatory for actin reorganization and cell migration [11, 22, 30, 36, 41, 43, 58], indicating another mechanism by which FIPI could impair cell migration.

In summary, FIPI potently blocked EGF-induced calcium release in MDA-NEO and MDA-HER2 human breast cancer cells. Although the mode of inhibition remains to be elucidated in ongoing studies, we conclude from our current data that FIPI most likely mediated PLD1 inhibition concomitant with lower PA and choline levels, resulting in weaker tyrosine phosphorylation-dependent PLC-γ1 activation and an overall decreased release of calcium from intracellular stores.

## Supplementary Information

**Additional file 2. Fig. S1**: Even low concentrations of FIPI significantly impaired EGF-induced calcium release in MDA-NEO and MDA-HER2 cells. Stimulation of cells with different concentrations of PA did not induce calcium release in either cell line, whereas EGF-induced calcium release was significantly blocked by different edelfosine concentrations. Shown are the mean ± S.E.M. of three independent experiments. Statistical analysis: one-way ANOVA and Tukey’s post hoc test: * = * p* < 0.05, ** = * p* < 0.01, *** = * p* < 0.001, **** = * p* < 0.0001.

**Additional file 3. Fig. S2**: Densitometric analysis of Western blot data are shown in Fig. 3a, c. The results are presented as the mean ± SEM. of at least four independent experiments. Statistics: EGFR, pEGFR, pHER2: Statistical analysis: one-way ANOVA and Kruskal–Wallis post hoc test: * = * p* < 0.05, ** = * p* < 0.01, *** = * p* < 0.001. pHER2: one-way ANOVA and Tukey post hoc test: * = * p* < 0.05.

**Additional file 4. Fig. S3**: Densitometric analysis of AKT, pAKT, MAPK and pMAPK Western blot data are shown in Fig. 3b, d. The mean ± SEM. of at least four independent experiments. Statistical analysis: one-way ANOVA and Kruskal–Wallis post hoc test: * = * p* < 0.05, ** = * p* < 0.01, *** = * p* < 0.001, **** = * p* < 0.0001.

**Additional file 5. Fig. S4**: Densitometric analysis of PLC-γ1 and pPLC-γ1 Western blot data are shown in Fig. 3b, d. The mean ± S.E.M. of at least four independent experiments. Statistical analysis using one-way ANOVA and the Kruskal–Wallis post hoc test revealed no statistical significance.

## Data Availability

The datasets used and/or analyzed during the current study are available from the corresponding author upon reasonable request.

## Abbreviations

AA: Arachidonic acid
BSA: Bovine serum albumin
cPLA_2_: Cyclic phospholipase A_2_
CSF-1: Colony stimulating factor-1
DAG: Diacylglycerol
EGF: Epidermal growth factor
EGFR: Epidermal growth factor receptor
FCS: Fetal calf serum
FIPI: 5-Fluoro-2-indolyl-des-chlorohalopemide
GPCR: G-protein coupled 7-transmembrane receptor
HER2: Human epidermal growth factor receptor 2
IP3: Inositol-1,4-5-triphosphate
IP3R: Inositol-1,4,5-trisphosphate receptor
MDA-HER2: MDA-MB-468-HER2
MDA-NEO: MDA-MB-468-NEO
MEM: Minimal essential medium
mTOR: Mammalian target of rapamycin
PA: Phosphatidic acid
PI3K: Phosphoinositide-3-kinase
PIP2: Phosphatidylinositol-4,5-bisphosphate
PIP3: Phosphatidylinositol-3,4,5-trisphosphate
PKC: Protein kinase C
PLC-γ1: Phospholipase C-γ1
PLD: Phospholipase D
PTK: Phosphotyrosine kinase
PVDF: Polyvinylidene difluoride
qPCR: Quantitative polymerase chain reaction
RTK: Receptor tyrosine kinase
S6K: S6-kinase
scRNA: Scrambled control RNA
SDS-PAGE: Sodium dodecyl sulphate–polyacrylamide gel electrophoresis
Sig-1R: Sigma-1 receptor
siRNA: Small interfering RNA
