# Supplementary figures and images for: The phospholipase D inhibitor FIPI potently blocks EGF-induced calcium signaling in human breast cancer cells
Source: Cell Commun Signal. 2021 Apr 8;19:43. doi: 10.1186/s12964-021-00724-z (PMC8034102; doi:10.1186/s12964-021-00724-z)

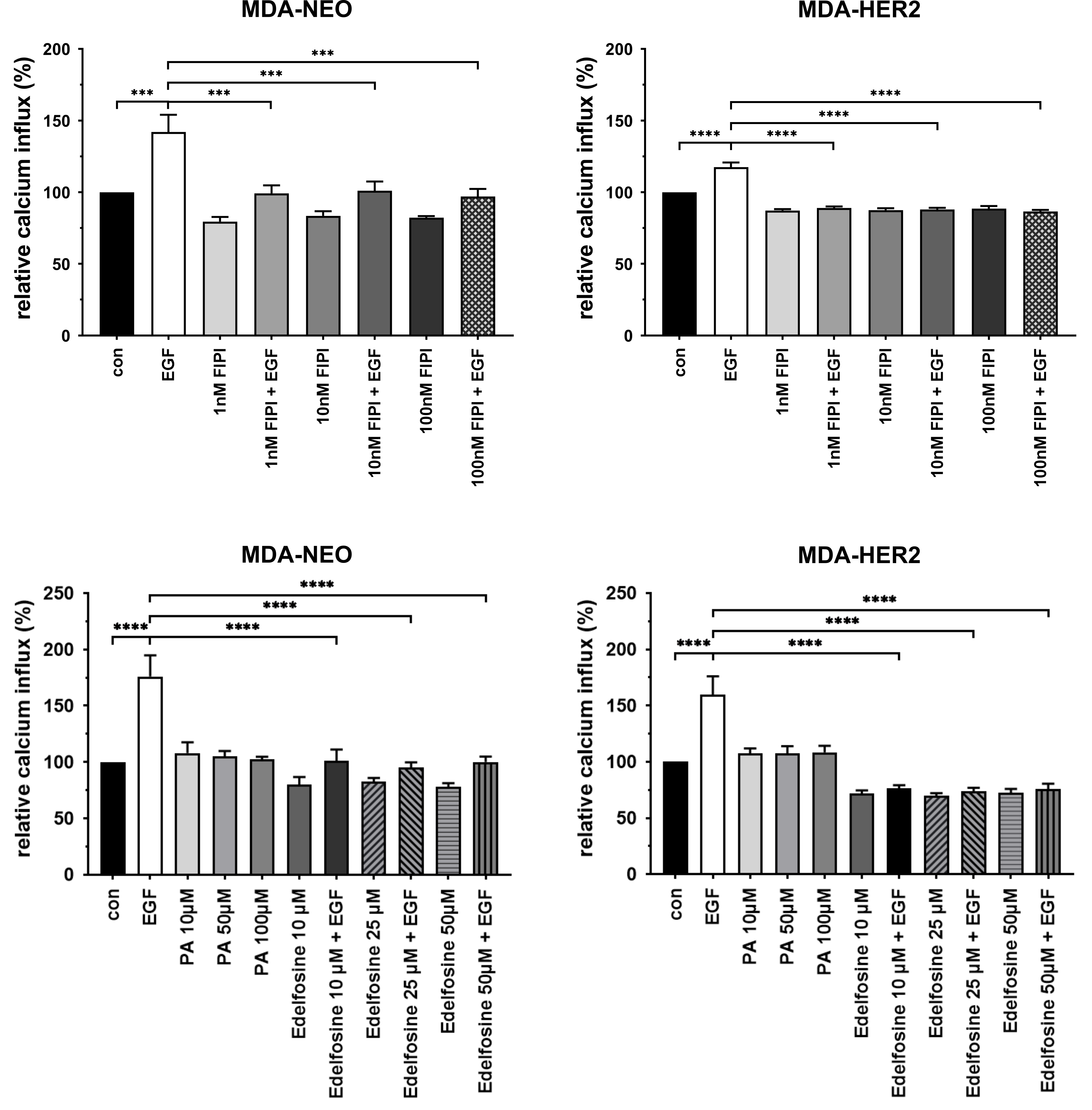

Supplement: Supplementary file 2 — Additional file 2. Fig. S1: Even low concentrations of FIPI significantly impaired EGF-induced calcium release in MDA-NEO and MDA-HER2 cells. Stimulation of cells with different concentrations of PA did not induce calcium release in either cell line, whereas EGF-induced calcium release was significantly blocked by different edelfosine concentrations. Shown are the mean ± S.E.M. of three independent experiments. Statistical analysis: one-way ANOVA and Tukey’s post hoc test: * = p < 0.05, ** = p < 0.01, *** = p < 0.001, **** = p < 0.0001. [file 12964_2021_724_MOESM2_ESM.png]

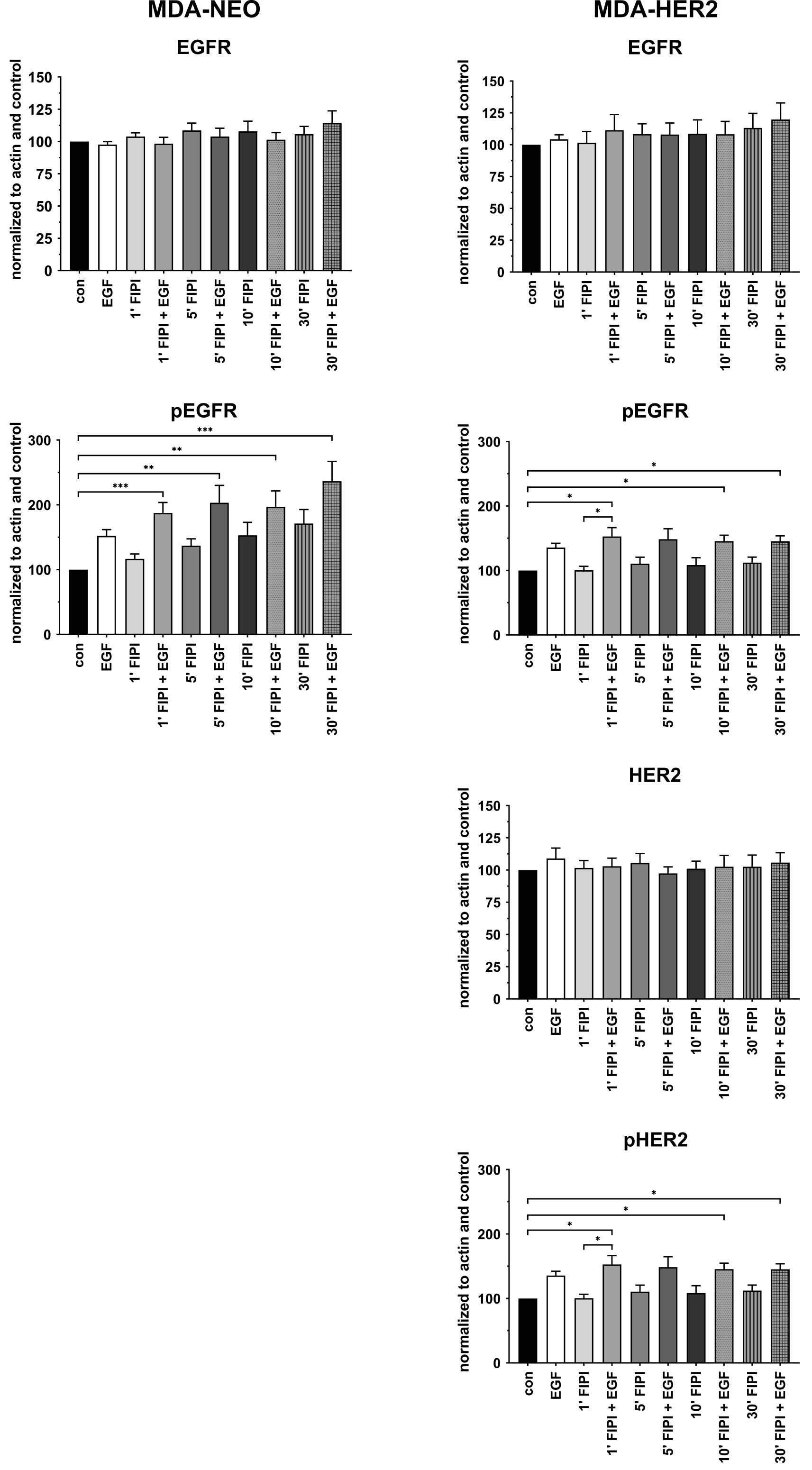

Supplement: Supplementary file 3 — Additional file 3. Fig. S2: Densitometric analysis of Western blot data are shown in Fig. 3a, c. The results are presented as the mean ± SEM. of at least four independent experiments. Statistics: EGFR, pEGFR, pHER2: Statistical analysis: one-way ANOVA and Kruskal–Wallis post hoc test: * = p < 0.05, ** = p < 0.01, *** = p < 0.001. pHER2: one-way ANOVA and Tukey post hoc test: * = p < 0.05. [file 12964_2021_724_MOESM3_ESM.png]

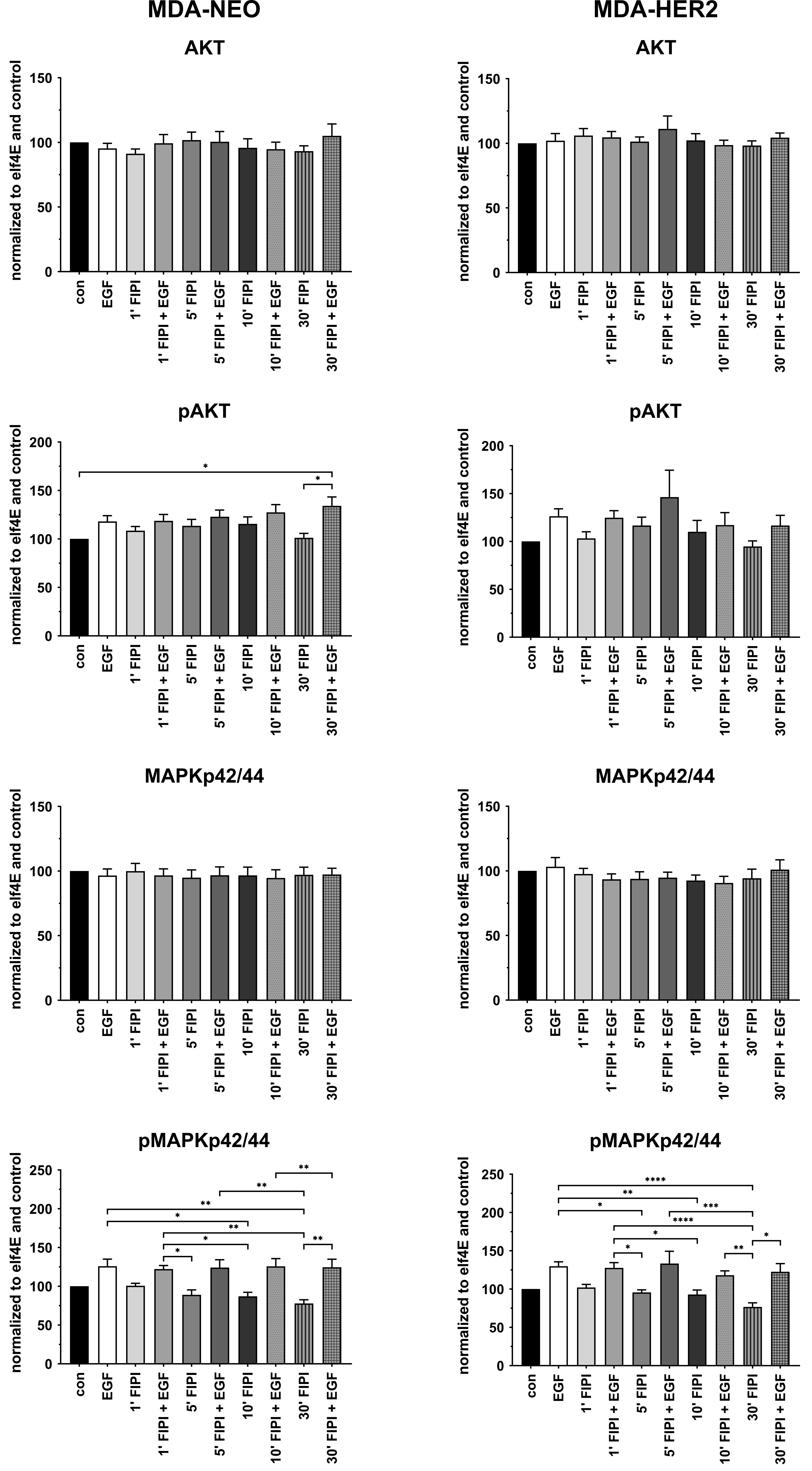

Supplement: Supplementary file 4 — Additional file 4. Fig. S3: Densitometric analysis of AKT, pAKT, MAPK and pMAPK Western blot data are shown in Fig. 3b, d. The mean ± SEM. of at least four independent experiments. Statistical analysis: one-way ANOVA and Kruskal–Wallis post hoc test: * = p < 0.05, ** = p < 0.01, *** = p < 0.001, **** = p < 0.0001. [file 12964_2021_724_MOESM4_ESM.png]

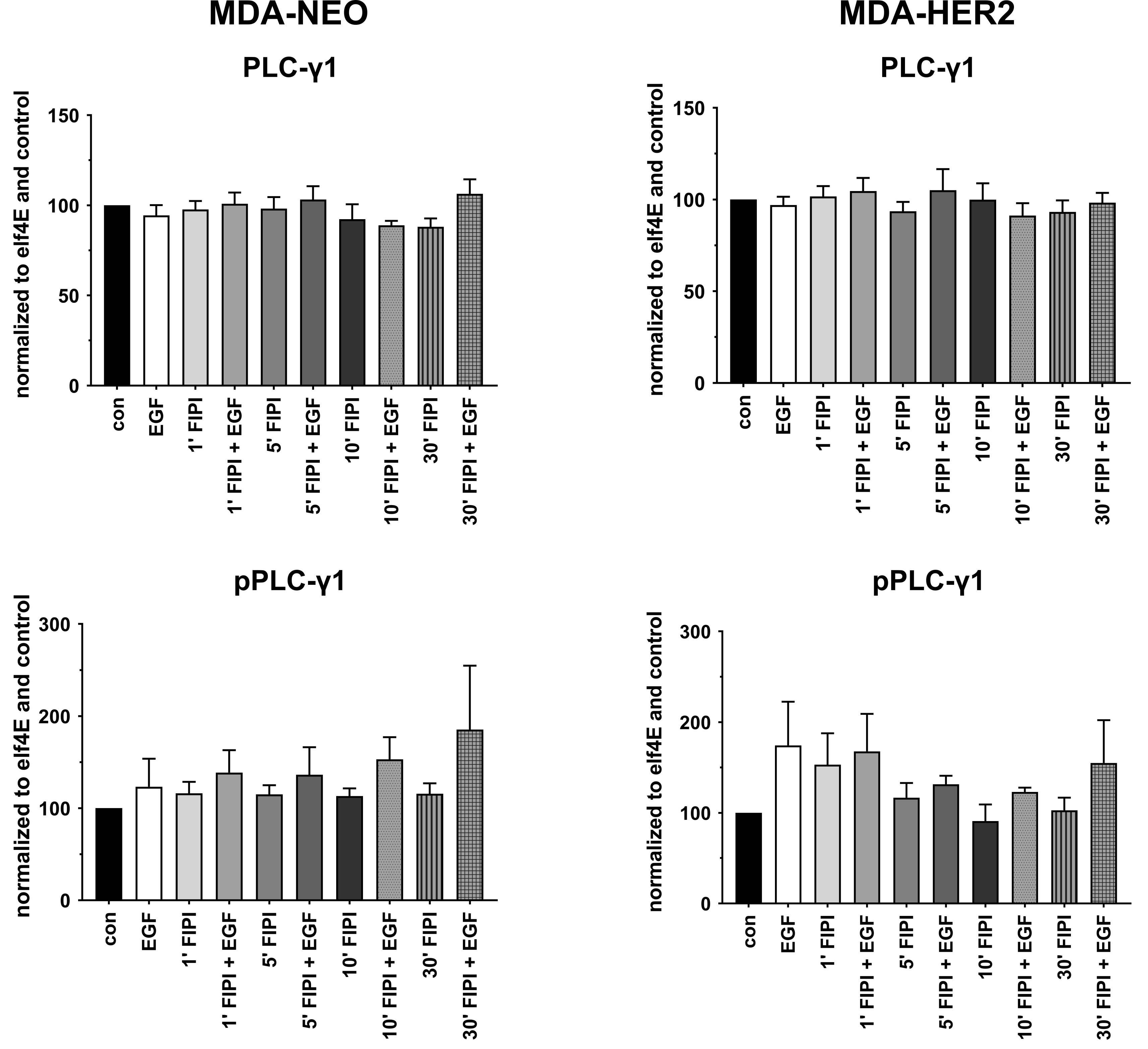

Supplement: Supplementary file 5 — Additional file 5. Fig. S4: Densitometric analysis of PLC-γ1 and pPLC-γ1 Western blot data are shown in Fig. 3b, d. The mean ± S.E.M. of at least four independent experiments. Statistical analysis using one-way ANOVA and the Kruskal–Wallis post hoc test revealed no statistical significance. [file 12964_2021_724_MOESM5_ESM.png]
